# A mortality prediction rule for non-elderly patients with community-acquired pneumonia

**DOI:** 10.1186/s12890-016-0199-z

**Published:** 2016-03-08

**Authors:** Masato Tashiro, Kiyohide Fushimi, Takahiro Takazono, Shintaro Kurihara, Taiga Miyazaki, Misuzu Tsukamoto, Katsunori Yanagihara, Hiroshi Mukae, Takayoshi Tashiro, Shigeru Kohno, Koichi Izumikawa

**Affiliations:** Department of Infectious Diseases, Nagasaki University Graduate School of Biomedical Sciences, 1-7-1 Sakamoto, Nagasaki, 852-8501 Japan; Department of Respiratory Diseases, Nagasaki University Graduate School of Biomedical Sciences, Nagasaki, Japan; Department of Health Sciences, Nagasaki University Graduate School of Biomedical Sciences, Nagasaki, Japan; Nagasaki University Infection Control and Education Centre, Nagasaki University Hospital, Nagasaki, Japan; Department of Laboratory Medicine, Nagasaki University Hospital, Nagasaki, Japan; Department of Health Informatics and Policy, Graduate School of Medicine, Tokyo Medical and Dental University, Tokyo, Japan

**Keywords:** Clinical prediction rule, Mortality, Pneumonia, Young adult, Middle aged

## Abstract

**Background:**

No mortality prediction rule is suited for non-elderly patients with community-acquired pneumonia. Therefore, we tried to create a mortality prediction rule that is simple and suitable for non-elderly patients with community-acquired pneumonia.

**Methods:**

Because of low mortality at young age, we used information from an administrative database that included A-DROP data. We analysed the rate and risk factors for in-hospital community-acquired pneumonia-associated death among non-elderly patients and created a mortality prediction rule based on those risk factors.

**Results:**

We examined 49,370 hospitalisations for patients aged 18–64 years with community-acquired pneumonia. The 30-day fatality rate was 1.5 %. Using regression analysis, five risk factors were selected: patient requires help for feeding, the existence of malignancy, confusion, low blood pressure, and age 40–64 years. Each risk factor of our proposed mortality risk scoring system received one point. A total point score for each patient was obtained by summing the points. The negative likelihood ratio for the score 0 group was 0.01, and the positive likelihood ratio for the score ≥4 group was 19.9. The area under the curve of the risk score for non-elderly (0.86, 95 % confidence interval: 0.84–0.87) was higher than that of the A-DROP score (0.72, 95 % confidence interval: 0.70–0.74) (*P* < 0.0001).

**Conclusions:**

Our newly proposed mortality risk scoring system may be appropriate for predicting mortality in non-elderly patients with community-acquired pneumonia. It showed a possibility of a better prediction value than the A-DROP and is easy to use in various clinical settings.

**Electronic supplementary material:**

The online version of this article (doi:10.1186/s12890-016-0199-z) contains supplementary material, which is available to authorized users.

## Background

Community-acquired pneumonia (CAP) remains a major cause of death for elderly and non-elderly people [1–4]. Particularly, in younger and middle-aged adults, most of whom are working-age adults, productivity losses due to death caused by CAP are an important problem from an economic and medical expenses standpoint [4]. It is also important to distinguish patients who are at low risk to prevent over-treatment, because there is a tendency to overestimate the risk of death in patients with CAP [5]. However, few studies have attempted to distinguish low-risk CAP patients from high risk ones and determine which patients would benefit when treated as outpatients. Because of these reasons, a precise prediction rule for identifying the severity of CAP is required, especially for non-elderly patients.

There are various indexes for estimating the severity of CAP patients [5–13]. Of those, the most common is the pneumonia severity index (PSI) and the CURB-65 score [9]. However, it is unknown whether both can correctly estimate the severity of CAP in non-elderly patients, because in previous studies, most patients have been elderly [14–19]. Therefore, our study aimed to create a mortality prediction rule suitable for non-elderly patients with CAP. Because of low morbidity and mortality at young age, we used information from an administrative database that included A-DROP data.

## Methods

### Data source and patient selection

We used a nationwide dataset available through the Japanese Diagnosis Procedure Combination (DPC) system [20]. This dataset was collected by our survey of DPC hospitals, which voluntarily participated with non-disclosure agreement. Public access to the DPC database was not permitted because it was private database of our study group. The DPC database includes claims and abstract discharge data for all inpatients discharged from >1,000 participating hospitals in Japan. We anonymised the data used upon extraction from the DPC database and analysed it within the protected environment of the Nagasaki University Hospital. Informed consent was not required for this study, and the institutional review board of the Nagasaki University Hospital (Nagasaki, Japan) approved the study design (Institutional Review Board No. 15022334).

The present study used data collected from January 2010 to December 2012. Patients’ ages were 18–64 years [2]. CAP was defined as the final diagnosis at hospital discharge by the International Classification of Diseases, tenth revision codes (ICD-10): J10-J18 (pneumonia) and J69 (aspiration pneumonia) [2]. We excluded patients who had any missing data from the statistical analyses.

### Data extraction

The following characteristics for each patient were extracted: age; sex; height; weight; final diagnosis; comorbidities on admission with ICD-10 codes; smoking status; Barthel index at admission, including the results of each item; pneumonia severity; length of stay; and death occurring within 30 days of hospital admission. In this study, there were no data after patients’ discharge. Comorbidities were evaluated using the Charlson index and the presence of each item [21]. The Charlson index includes seventeen conditions with major impact on survival described as Table 1. For each condition, between one and six points are awarded and summed up for the summary score. Higher score indicate severe comorbidities [22]. The Barthel index is a 10-item measure of activities, such as feeding, moving from a wheelchair to bed and back, personal grooming (washing and shaving face, and combing hair), transferring to and from a toilet, bathing (patient can use a bath tub, a shower, or take a complete sponge bath), walking on a level surface, ascending and descending stairs, dressing, and controlling bowels and bladder [23, 24]. Those were scored according to assistance required by the patient (Additional file 1: Table S1). The total score was from 0–100 with lower scores representing greater nursing dependency. The pneumonia severity was evaluated using the A-DROP system, which is the modified CURB-65 scoring system proposed by The Japanese Respiratory Society. It assesses Age, Dehydration (existence of a clinical sign of dehydration or blood urea nitrogen level ≥210 mg/L), Respiratory failure (SpO_2_ ≤ 90 % or PaO_2_ ≤ 60 mmHg), Orientation disturbance (confusion), and a low blood Pressure (systolic blood pressure ≤90 mmHg) [25]. The scoring system stratifies patients into four severity classes (mild = 0; moderate = 1–2; severe = 3; and extremely severe = 4–5), and it has an equal ability for predicting the mortality of CAP compared to the CURB-65 scoring system [6]. In this study, the score for age was 0 points in all patients because all were <65 years.

**Table 1:** Baseline characteristics and mortality of the study cohort

Characteristic	No. of cases (%)	No. (%) of cases alive at 30 days	No. (%) of cases that died within 30 days
	(*n* = 49,370)	(*n* = 48,638)	(*n* = 732)
Sex			
Male	28,070 (56.9)	27,550 (56.6)	520 (71.0)
Female	21,300 (43.1)	21,088 (43.4)	212 (29.0)
Age, years			
18–39	14,791 (30.0)	14,753 (30.3)	38 (5.2)
40–49	7,167 (14.5)	7,106 (14.6)	61 (8.3)
50–64	27,412 (55.5)	26,779 (55.1)	633 (86.5)
Body mass index (kg/m^2^)			
≤ 15	2,485 (5.0)	2,360 (4.9)	125 (17.1)
16–29	44,419 (90.0)	43,834 (90.1)	585 (79.9)
≥ 30	2,466 (5.0)	2,444 (5.0)	22 (3.0)
Smoking status			
Never smoked	27,883 (56.5)	27,506 (56.6)	377 (51.5)
Current/ex-smoker	21,487 (43.5)	21,132 (43.4)	355 (48.5)
Charlson comorbidity index			
0–1	37,642 (76.2)	37,269 (76.6)	373 (51.0)
2–4	10,534 (21.3)	10,270 (21.1)	264 (36.1)
≥ 5	1,194 (2.4)	1,099 (2.3)	95 (13.0)
Myocardial infarction	330 (0.7)	324 (0.7)	6 (0.8)
Congestive heart failure	2,667 (5.4)	2,572 (5.3)	95 (13.0)
Peripheral vascular disease	264 (0.5)	259 (0.5)	5 (0.7)
Cerebrovascular diseases	1,497 (3.0)	1,458 (3.0)	39 (5.3)
Dementia	201 (0.4)	192 (0.4)	9 (1.2)
Chronic pulmonary disease	8,503 (17.2)	8,426 (17.3)	77 (10.5)
Rheumatic disease	1,854 (3.8)	1,818 (3.7)	36 (4.9)
Peptic ulcer disease	1,910 (3.9)	1,886 (3.9)	24 (3.3)
Mild liver disease	2,464 (5.0)	2,435 (5.0)	29 (4.0)
Diabetes without chronic complications	4,741 (9.6)	4,647 (9.6)	94 (12.8)
Diabetes with chronic complications	1,587 (3.2)	1,547 (3.2)	40 (5.5)
Hemiplegia or paraplegia	154 (0.3)	153 (0.3)	1 (0.1)
Renal disease	1,490 (3.0)	1,444 (3.0)	46 (6.3)
Any malignancy	4,364 (8.8)	4,218 (8.7)	146 (19.9)
Moderate or severe liver disease	61 (0.1)	56 (0.1)	5 (0.7)
Metastatic solid tumour	860 (1.7)	777 (1.6)	83 (11.3)
AIDS/HIV	81 (0.2)	80 (0.2)	1 (0.1)
Barthel index (score)			
81–100	37,035 (75.0)	36,848 (75.8)	187 (25.5)
41–80	4,733 (9.6)	4,644 (9.5)	89 (12.2)
0–40	7,602 (15.4)	7,146 (14.7)	456 (62.3)
Requires help with			
Feeding	8,928 (18.1)	8,448 (17.4)	480 (65.6)
Moving from the wheelchair to the bed	12,367 (25.0)	11,832 (24.3)	535 (73.1)
Personal grooming	9,842 (19.9)	9,345 (19.2)	497 (67.9)
Getting on/off toilet	10,869 (22.0)	10,345 (21.3)	524 (71.6)
Bathing	12,610 (25.5)	12,065 (24.8)	545 (74.5)
Walking on a level surface	12,581 (25.5)	12,035 (24.7)	546 (74.6)
Ascending and descending stairs	13,433 (27.2)	12,872 (26.5)	561 (76.6)
Dressing	12,572 (25.5)	12,032 (24.7)	540 (73.8)
Controlling the bowels	8,313 (16.8)	7,844 (16.1)	469 (64.1)
Controlling the bladder	8,377 (17.0)	7,906 (16.3)	471 (64.3)

Definition of abbreviations: *AIDS* acquired immune deficiency syndrome, *HIV* human immunodeficiency virus

In addition to patients’ characteristics, we extracted other factors that may be associated with mortality such as health care region organisational factors, which included the hospital volume, distance from patients’ home to the hospital, weekend or holiday admission, and intensive care unit admission [26–29].

### Statistical analyses

We used standard deviations in our analyses. To examine differences in characteristics among groups, Fisher’s exact test or Pearson’s chi-square test was used to analyse discrete variables, and the Wilcoxon rank sum test was used for continuous variables. The health care region organisational factors were examined for association with CAP mortality by using univariable and multivariable logistic regression analyses. Odds ratios (ORs) and their 95 % confidence intervals (CIs) were calculated. Statistical analyses were performed using JMP 11.2 software (SAS Institute, Cary, NC, USA). All tests were two-tailed, and a *P*-value <0.05 was considered statistically significant.

To determine the order of importance of factors predicting 30-day case fatality, we conducted a forward stepwise selection of the factors. The sources of selected factors were the demographics, comorbidities, Barthel index items, parameters of the A-DROP (confusion, dehydration, respiratory failure, and low blood pressure), and health care region organisation. Scores of the Charlson comorbidity and Barthel indices were excluded; alternatively, a particular item of each index was used because calculation of those indexes is difficult in busy clinical settings. Based on the stepwise selection of factors and from a standpoint of ease of use, we selected five items for predicting 30-day mortality. Each risk factor of our proposed mortality risk scoring system received one point. A total point score for each patient was obtained by summing the points. The validity of the new prediction rule compared to the A-DROP system for CAP mortality was evaluated using the receiver operating characteristics (ROC) curve, Kaplan-Meier survival curves, and log-rank analyses. Overall, the model prediction was expressed as a c statistic [2, 30].

## Results

### Baseline characteristics and mortality

There were 77,819 hospitalised patients with CAP, among which 28,849 patients’ data were incomplete. Therefore, we analysed the remaining 49,370 hospitalisations of patients aged 18–64 years with CAP. Table 1 shows the baseline characteristics. The 30-day case-fatality rate was 1.5 %. The percentage of patients who died was higher among men than women (*P* < 0.0001). An older age, high Charlson comorbidity index, low Barthel index, and high severity of pneumonia were associated with a higher mortality (*P* < 0.0001, all). Body mass index was also associated with CAP mortality (*P* < 0.0001), particularly in thin patients with a body mass index <15 kg/m^2^ (30-day mortality: 5.0 %).

As observed in the pneumonia aetiology, aspiration pneumonia had the highest in-hospital mortality (4.5 %), followed by influenza pneumonia (1.8 %) (Table 2). The lowest mortality was observed for mycoplasmal pneumonia (0.1 %).

**Table 2:** Pneumonia characteristics and mortality

Characteristic	No. of cases (%)	No. (%) of cases alive at 30 days	No. (%) of cases that died within 30 days
	(*n* = 49,370)	(*n* = 48,638)	(*n* = 732)
Parameters of the A-DROP^a^			
Confusion	927 (1.9)	832 (1.7)	95 (13.0)
Dehydration	3,390 (6.9)	3,255 (6.7)	135 (18.4)
Respiratory failure	3,940 (8.0)	3,779 (7.8)	161 (22.0)
Low blood pressure	6,697 (13.6)	6,353 (13.1)	344 (47.0)
Pneumonia severity^b^			
Mild	37,094 (75.1)	36,845 (75.8)	249 (34.0)
Moderate	11,712 (23.7)	11,308 (23.2)	404 (55.2)
Severe	411 (0.8)	367 (0.8)	44 (6.0)
Extremely severe	153 (0.3)	118 (0.2)	35 (4.8)
Pneumonia aetiology			
Pneumococcal	4,392 (8.9)	4,361 (9.0)	31 (4.2)
Mycoplasmal	5,010 (10.1)	5,004 (10.3)	6 (0.8)
Aspiration	3,586 (7.3)	3,425 (7.0)	161 (22.0)
Influenza	167 (0.3)	164 (0.3)	3 (0.4)
Others^c^	36,215 (73.4)	35,684 (73.4)	531 (72.5)

^a^A-DROP assesses confusion, dehydration (the existence of a clinical sign of dehydration or blood urea nitrogen level ≥210 mg/L), respiratory failure (SpO_2_ ≤ 90 % or PaO_2_ ≤ 60 mmHg), and a low blood pressure (systolic blood pressure ≤90 mmHg). Age was excluded from this table because all patients were <65 years

^b^The pneumonia severity was evaluated using the A-DROP scoring system. Scoring: 0 = mild, 1–2 = moderate, 3 = severe, and 4 = extremely severe

^c^This group includes other bacterial or viral and unspecified bacterial pneumonia

### Health care region organisational factors

Table 3 shows the association between the CAP mortality of non-elderly patients and the health care region organisational factors. As observed in the results of crude ORs, the risk of 30-day mortality was higher in patients who were admitted to a high volume hospital, whose homes were far from the hospital, and who were admitted on weekends or holidays. However, the pneumonia severity was also higher in patients with those factors. Therefore, the adjusted ORs showed no significant difference for those factors.

**Table 3:** Health care region organisational factors

		30-day mortality	
Factor	No. of cases (%)	Crude OR	Adjusted OR^a^
	(*n* = 49,370))	(95 % CI)	(95 % CI)
Hospital volume (no. of beds)			
≤ 200	7,373 (14.9)	Reference	Reference
201–600	31,868 (64.5)	1.2 (1.0–1.5)	1.0 (0.8–1.3)
≥ 601	10,129 (20.5)	1.7 (1.4–2.3)	1.2 (0.9–1.5)
Distance from the patients’ home to the hospital (km)			
≤10	14,325 (29.0)	Reference	Reference
11–30	23,782 (48.2)	1.3 (1.1–1.5)	0.9 (0.8–1.1)
≥31	11,263 (22.8)	1.6 (1.3–1.9)	1.0 (0.8–1.2)
Weekend or holiday admission	10,237 (20.7)	1.3 (1.1–1.5)	1.1 (0.9–1.3)

Definition of abbreviations: *OR* odds ratio, *CI* confidence interval

^a^The ORs were adjusted for all factors of patients’ characteristics, pneumonia characteristics, and health care region organisational factors. The c statistic for the model is 0.89217

### Newly proposed mortality prediction rule

Stepwise analysis selected the following factors for predicting 30-day mortality: age 40–49 years, age 50–64 years, body mass index ≤15 kg/m^2^, congestive heart failure, cerebrovascular diseases, any malignancy, metastatic solid tumour, patients requiring help with feeding, patients requiring help ascending and descending stairs, control of the bowels, respiratory failure, confusion, and low blood pressure. Among them, we carefully selected five factors in terms of ease of use and degree of significance for predicting mortality, which included patients requiring help with feeding, the existence of malignancy (any malignancy including metastatic solid tumour), confusion, low blood pressure, and age 40–64 years.

Patients requiring help with feeding were unable to eat, needed assistance cutting food, spreading butter, etc., and required a modified diet. Little is known about the association between CAP mortality and independent feeding in CAP patients. Therefore, we compared characteristics between patients who were independent and those who required help with feeding (Table 4). Patients who required help with feeding were predominately male, were thinner, had a high Charlson comorbidity index, and had a significant lower activity than those who were independent with feeding (*P* < 0.0001, all). Interestingly, aspiration pneumonia was a significantly predominant aetiology in patients who required help with feeding (*P* < 0.0001). In addition, these patients’ pneumonia was more severe, and they stayed in the hospital longer and had a high mortality (*P* < 0.0001, both).

**Table 4:** A comparison of patients’ characteristics with and without help feeding

	Feeding	
	Independent	Requires help
Characteristic	(*n* = 40,442)	(*n* = 8,928)
Personal characteristics		
Female sex	18,194 (45.0)	3,106 (34.8)
Age, mean years ± SD	47.8 ± 13.8	50.2 ± 14.3
Body mass index, mean ± SD	22.0 ± 4.5	19.8 ± 5.4
Charlson comorbidity index, mean ± SD	0.9 ± 1.3	1.0 ± 1.3
Barthel index, mean ± SD	96.1 ± 11.6	15.1 ± 23.1
Pneumonia severity^a^		
Mild	31,983 (79.1)	5,111 (57.3)
Moderate	8,239 (20.4)	3,473 (38.9)
Severe	175 (0.4)	236 (2.6)
Extremely severe	45 (0.1)	108 (1.2)
Pneumonia aetiology^b^		
Pneumococcal	3,789 (9.4)	603 (6.8)
Mycoplasmal	4,770 (11.8)	240 (2.7)
Aspiration	758 (1.9)	2,828 (31.7)
Influenza	137 (0.3)	30 (0.3)
Outcome after hospitalisation		
Died within 30 days	252 (0.6)	480 (5.4)
Length of stay (days), mean ± SD	11.3 ± 14.0	26.2 ± 61.1
ICU admission	229 (0.6)	439 (4.9)

Definition of abbreviations: *SD* standard deviation, *ICU* intensive care unit

Data are n (%) of patients, unless noted otherwise

^a^The pneumonia severity was evaluated using the A-DROP scoring system, which assesses confusion, dehydration (the existence of a clinical sign of dehydration or blood urea nitrogen level ≥210 mg/L), respiratory failure (SpO_2_ ≤ 90 % or PaO_2_ ≤ 60 mmHg), and a low blood pressure (systolic blood pressure ≤90 mmHg). Scoring: 0 = mild, 1–2 = moderate, 3 = severe, and 4 = extremely severe

^b^The percentages are not equal to 100 % because other bacterial or viral and unspecified bacterial pneumonia are excluded from this table

### Validation of the prediction rule

**Fig. 1:**
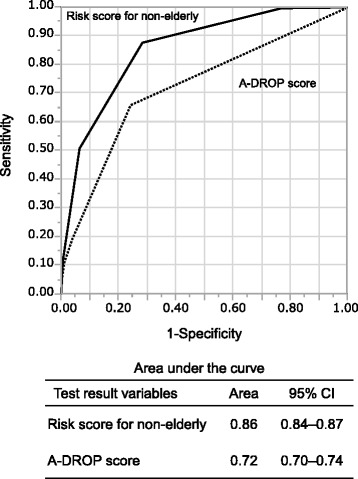
Receiver operating characteristic curves for predicting 30-day mortality of non-elderly patients with community-acquired pneumonia. The risk score for non-elderly (range, 0–5) is calculated by summing the existence of the following characteristics: patient requires help with feeding; the existence of a malignancy (any malignancy including metastatic solid tumour); confusion; low blood pressure (systolic blood pressure ≤90 mmHg), and age 40–64 years. The A-DROP score (range, 0–5) is obtained by summing the existence of the following characteristics: confusion, dehydration (existence of a clinical sign of dehydration or blood urea nitrogen level ≥210 mg/L), respiratory failure (SpO_2_ ≤ 90 % or PaO_2_ ≤ 60 mmHg), a low blood pressure (systolic blood pressure ≤90 mmHg), and age. For A-DROP, scores for age are 0 points in all patients because all are <65 years. The area under the curve of the risk score for non-elderly (0.86, 95 % confidence interval [CI]: 0.84–0.87) is higher than that of the A-DROP score (0.72, 95 % CI: 0.70–0.74) (*P* < 0.0001)

Table 5 shows regression results for 30-day death among non-elderly patients with CAP using parameters of the A-DROP and the newly proposed five risk factors. Most ORs of the proposed score parameters were higher than that of the A-DROP parameters. Figure 1 shows the ROC curves for the new mortality risk score and A-DROP score for non-elderly patients. The area under the curve (AUC) for our method of predicting 30-day mortality was 0.86 (95 % CI: 0.84–0.87). It was higher than the AUC for the A-DROP score, which was 0.72 (95 % CI: 0.70–0.74) (*P* < 0.0001).

**Table 5:** Logistic regression for all non-elderly patients who died within 30 days

Factor	OR (95 % CI)^a^
Parameters of A-DROP^b^	
Confusion	2.9 (2.1–3.9)
Dehydration^c^	1.6 (1.3–2.1)
Respiratory failure^d^	2.1 (1.7–2.6)
Low blood pressure^e^	5.5 (4.7–6.4)
Parameters of the risk score for non-elderly patients^f^	
Requires help with feeding	7.1 (6.0–8.4)
Malignancy^g^	4.3 (3.6–5.0)
Confusion	3.0 (2.4–3.9)
Low blood pressure^e^	3.3 (2.8–3.9)
Age group, 40–64 years^h^	4.7 (3.4–6.7)

Definition of abbreviations: *OR* odds ratio, *CI* confidence interval

^a^The ORs were adjusted for parameters of the A-DROP or risk score for non-elderly patients

^b^Age was excluded from this table because all patients were <65 years. The c statistic for the model is 0.73273

^c^The existence of a clinical sign of dehydration or blood urea nitrogen level ≥210 mg/L

^d^SpO_2_ ≤ 90 % or PaO_2_ ≤ 60 mmHg

^e^Systolic blood pressure ≤90 mmHg

^f^The c statistic for the model is 0.86673

^g^Any malignancy including metastatic solid tumour

^h^The reference group is 18–39 years

**Fig. 2:**
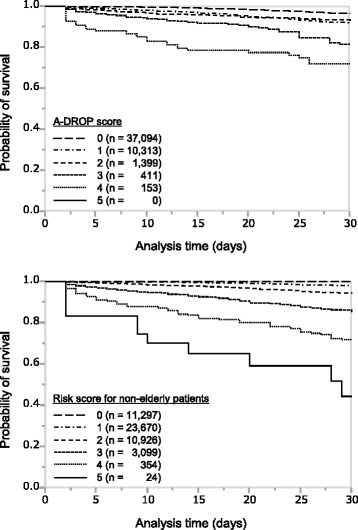
Kaplan-Meier survival curves classified by the A-DROP score and the risk score for non-elderly. The A-DROP score (range, 0–5) is obtained by summing the existence of the following characteristics: confusion, dehydration (existence of a clinical sign of dehydration or blood urea nitrogen level ≥210 mg/L), respiratory failure (SpO_2_ ≤ 90 % or PaO_2_ ≤ 60 mmHg), a low blood pressure (systolic blood pressure ≤90 mmHg), and age. For A-DROP, scores for age are 0 points in all patients because all are <65 years. The risk score for non-elderly (range, 0–5) is calculated by summing the existence of the following characteristics: patient requires help with feeding; the existence of malignancy (any malignancy including metastatic solid tumour); confusion; low blood pressure (systolic blood pressure ≤90 mmHg), and age 40–64 years. In both survival curves, there are significant differences for each group (*P* < 0.0001, all)

We also performed a comparison of the Kaplan-Meier survival curves, as classified by the A-DROP score and the new mortality risk score for non-elderly patients (Fig. 2). In both survival curves, there were significant differences between groups (*P* < 0.0001, both). Our method identified groups that would not die in the hospital compared to the A-DROP system, as observed in comparison between the score 0 group of our method and the score 0 group of the A-DROP score. Regarding the ability to detect patients who would die in the hospital, the proposed risk score more accurately detected a group with high mortality than the A-DROP score. In addition, the new risk score more clearly divided each group with different mortalities because margins of mortality between each group were larger in risk scores than in A-DROP scores for non-elderly patients.

Table 6 shows patients’ characteristics for each mortality risk score. There was a significant relationship between the risk score and various characteristics, excluding influenza pneumonia (almost *P* < 0.0001; influenza pneumonia, *P* = 0.9158). Particularly, few patients died (only 2 died in 30 days) among 11,297 patients in the lowest risk score (0). The negative likelihood ratio of the score 0 group was 0.01 (95 % CI: 0.00–0.04) for 30-day death. The positive likelihood ratio was 19.9 (95 % CI: 15.8–24.9) for the score ≥4 group.

**Table 6:** Comparison of characteristics in each group of risk score for non-elderly patients with community-acquired pneumonia

	Risk score for non-elderly patients^a^					
Characteristic	0	1	2	3	4	5
No. of patients	11,297 (100.0)	23,670 (100.0)	10,926 (100.0)	3,099 (100.0)	354 (100.0)	24 (100.0)
Personal characteristics						
Female sex	5,928 (52.5)	10,464 (44.2)	3,929 (36.0)	870 (28.1)	101 (28.5)	8 (33.3)
Age, mean years ± SD	30.0 ± 6.3	51.9 ± 11.4	56.0 ± 8.7	57.9 ± 6.6	59.1 ± 5.3	59.5 ± 5.5
Body mass index (kg/m^2^), mean ± SD	21.9 ± 4.7	21.9 ± 4.8	21.2 ± 4.9	20.1 ± 4.6	19.7 ± 4.3	18.1 ± 3.9
Charlson comorbidity index, mean ± SD	0.3 ± 0.6	0.7 ± 0.9	1.7 ± 1.6	1.9 ± 1.8	2.5 ± 2.0	3.5 ± 1.6
Barthel index, mean ± SD	97.7 ± 8.0	89.7 ± 25.4	63.1 ± 42.1	31.6 ± 38.9	16.4 ± 25.2	7.5 ± 16.5
Pneumonia severity^b^						
Mild	10,485 (92.8)	19,996 (84.5)	6,294 (57.6)	319 (10.3)	0 (0.0)	0 (0.0)
Moderate	812 (7.2)	3,652 (15.4)	4,500 (41.2)	2,554 (82.4)	194 (54.8)	0 (0.0)
Severe	0 (0.0)	22 (0.1)	121 (1.1)	195 (6.3)	67 (18.9)	6 (25.0)
Extremely severe	0 (0.0)	0 (0.0)	11 (0.1)	31 (1.0)	93 (26.3)	18 (75.0)
Pneumonia aetiology^c^						
Pneumococcal	897 (7.9)	2,345 (9.9)	878 (8.0)	246 (7.9)	25 (7.1)	1 (4.2)
Mycoplasmal	2,813 (24.9)	1,799 (7.6)	362 (3.3)	35 (1.1)	1 (0.3)	0 (0.0)
Influenza	41 (0.4)	79 (0.3)	33 (0.3)	12 (0.4)	2 (0.6)	0 (0.0)
Aspiration	59 (0.5)	860 (3.6)	1,720 (15.7)	855 (27.6)	86 (24.3)	6 (25.0)
Outcome after hospitalisation						
Died within 30 days	2 (0.0)	88 (0.4)	270 (2.5)	285 (9.2)	76 (21.5)	11 (45.8)
Length of stay (days), mean ± SD	8.0 ± 5.6	12.1 ± 17.7	18.8 ± 29.1	30.2 ± 70.9	37.3 ± 155.3	29.2 ± 36.6
ICU admission	30 (0.3)	160 (0.7)	263 (2.4)	181 (5.8)	33 (9.3)	1 (4.2)

Definition of abbreviations: *SD* standard deviation

Data are n (%) of patients, unless noted otherwise

^a^Each risk factor (requires help with feeding, malignancy, confusion, low blood pressure, and age 40–64) receives one point. A total point score for each patient is obtained by summing the points

^b^The pneumonia severity was evaluated using the A-DROP scoring system, which assesses confusion, dehydration (the existence of a clinical sign of dehydration or blood urea nitrogen level ≥210 mg/L), respiratory failure (SpO_2_ ≤ 90 % or PaO_2_ ≤ 60 mmHg), and a low blood pressure (systolic blood pressure ≤90 mmHg). Scoring: 0 = mild, 1–2 = moderate, 3 = severe, and 4 = extremely severe

^c^The percentages are not equal to 100 % because other bacterial or viral and unspecified bacterial pneumonia are excluded from this table

## Discussion

Our new mortality risk scoring system had a greater ability for predicting death due to CAP in non-elderly adults. Importantly, this system is easy to use in busy clinical settings, and is based on patients’ independence with feeding, the existence of a malignancy, confusion, low blood pressure, and age. Clinicians can evaluate all of these parameters before conducting laboratory tests. The existence of a malignancy can be confirmed by patients’ medical history, even though we cannot evaluate its existence in a case on the initial visit. Regarding independence with feeding in patients with CAP, any medical staff can evaluate this quickly. Furthermore, the assigned points consist of only one, and only five factors have to be assessed, which simplify the mortality risk score calculation.

One of our study objectives was to create a new method to prevent unnecessary over-treatment in non-elderly patients with CAP who would survive. It has been reported that only the PSI may be superior at identifying low-risk patients because it achieved a negative likelihood ratio of <0.1, whereas the CURB-65 did not [14]. Generally, a negative likelihood ratio of <0.1 is regarded as necessary for a predictive or diagnostic test to be considered robust [31]. In our study, the score 0 group among the new risk scores for non-elderly patients achieved a negative likelihood ratio of <0.1, but the score ≤1 group did not. We found that patients in the score 0 group would not die in 30 days.

In our study cohort, less than 15 % of patients with severe disease according the risk score proposed were admitted to ICU. In addition, only one patient was admitted in ICU in highest severity of pneumonia (Table 6). The reason of a low ICU rate admission might be due to the fact that the number of ICU beds was smaller in Japan than other countries. Sasabuchi Y et al. reported an ICU-to-hospital bed ratio in their study was relatively small compared with those in Western countries [32]. Therefore some patients with severe disease in Japan were obliged to admit to non-ICU beds.

In a similar study from Canada, Marrie et al. referenced a limitation to their study [2]. They mentioned that their study findings may not be generalisable to other countries. Despite the fact that our study was conducted in Japan, most of the findings were similar to theirs. We confirmed that age, sex, and comorbidity were significantly associated with death in non-elderly patients, and patients with aspiration pneumonia had a high mortality. In addition, larger hospitals had higher case-fatality rates, but this was more likely related to greater comorbidities and the severity of pneumonia. Therefore, these features among non-elderly patients may be similar worldwide. Another limitation to their study was that they did not use the data from a common severity index such as the PSI or CURB-65. We were able to perform our analyses more accurately since we used the A-DROP score.

There are several limitations to our study. First, we could not compare the predictive ability of our new mortality risk scoring system to that of the PSI. To detect low-risk patients, a non-inferiority comparison between our system and the PSI is needed. Second, a validation cohort such as an outpatient population should confirm this model in the future. Third, there may be several factors associated with mortality that were not considered in this study. For example, our study did not include factors such as the results of laboratory tests, information on drugs used to suppress the immune system, the use of antibacterial agents before admission, a history of hospital admissions, or socioeconomic status. The income of each individual, which was not included in this study, would never affect the type of treatment because of the universal health insurance coverage in Japan [33]. Fourth, we could not distinguish healthcare-associated pneumonia from CAP, of which the clinical profile is different from that of CAP [34]. Most patients who died may be categorised as healthcare-associated pneumonia because the analysis showed that the existence of a malignancy and reduction of Activities of Daily Living were important risk factors. Fifth, there were several limitations to the administrative databases. Rothberg et al. reported that variation in the use of the principal diagnosis of sepsis or respiratory failure might bias efforts to compare hospital performance regarding pneumonia outcomes [35]. We may have missed severe pneumonia patients with sepsis or respiratory failure because the same phenomenon could have occurred in this study.

## Conclusion

Our newly proposed mortality risk scoring system may be appropriate for predicting the mortality of non-elderly patients with CAP. It showed the possibility of a better prediction value than the A-DROP. Additionally, it was easy to use in various clinical settings. Clinicians should provide these patients with appropriate treatment according to a precise mortality prediction.

## Additional file

Additional file 1: Table S1. Definition of scores for each item of the Barthel index used in this study. (DOCX 49.2kb)

## Abbreviations

CAP: Community-acquired pneumonia
PSI: Pneumonia severity index
DPC: Japanese diagnosis procedure combination
ICD-10: International classification of diseases, tenth revision codes
ORs: Odds ratios
Cis: Confidence intervals
ROC: Receiver operating characteristics
