# Supplementary material for: A mortality prediction rule for non-elderly patients with community-acquired pneumonia
Source: BMC Pulm Med. 2016 Mar 8;16:39. doi: 10.1186/s12890-016-0199-z (PMC4784337; doi:10.1186/s12890-016-0199-z)
Supplement: Additional file 1: Table S1. — Definition of scores for each item of the Barthel index used in this study. (DOCX 49.2kb) [file 12890_2016_199_MOESM1_ESM.docx]

| **Table S1. Definition of scores for each item of the Barthel index used in this study** | | | |
| --- | --- | --- | --- |
| **Feeding** | | **Walking on a level surface** | |
| 0 = | unable | 0 = | immobile |
| 5 = | need help cutting, spreading butter, etc. | 5 = | wheelchair independent |
| 10 = | independent | 10 = | walks with the help of one person |
|  |  | 15 = | independent |
| **Moving from the wheelchair to the bed** | | **Ascending and descending stairs** | |
| 0 = | Unable, no sitting balance | 0 = | unable |
| 5 = | major help, can sit | 5 = | needs help |
| 10 = | minor help | 10 = | independent |
| 15 = | independent |  |  |
| **Personal grooming (wash and shave face, and comb hair)** | | **Dressing** | |
| 0 = | needs help with personal care | 0 = | dependent |
| 5 = | independent face washing/hair brushing/teeth cleaning/shaving | 5 = | needs help |
|  |  | 10 = | independent |
| **Getting on/off toilet** | | **Controlling the bowels** | |
| 0 = | dependent | 0 = | incontinent |
| 5 = | needs some help, but can do something alone | 5 = | occasional accident |
| 10 = | independent | 10 = | continent |
| **Bathing** | | **Controlling the bladder** | |
| 0 = | dependent | 0 = | incontinent |
| 5 = | independent | 5 = | occasional accident |
|  |  | 10 = | continent |
